# An Effective Life–Sex–Emotions Course for Taiwanese Adolescents on Health Perceptions: A Cohort Study

**DOI:** 10.3389/fpubh.2021.660229

**Published:** 2021-05-19

**Authors:** Kuo-Yu Chao, Wei Cheng

**Affiliations:** ^1^Department of Nursing, Chang Gung University of Science and Technology, Taoyuan, Taiwan; ^2^Division of Colon and Rectal Surgery, Chang Gung Memorial Hospital, Linkou, Taiwan; ^3^Department of Pathology, Kee-Lung Hospital, Ministry of Health and Welfare, Kee-Lung, Taiwan; ^4^School of Nursing, National Taipei University of Nursing and Health Sciences, Taipei, Taiwan; ^5^Department of Nursing, Ching Kuo Institute of Management and Health, Kee-Lung, Taiwan

**Keywords:** adolescence, life-sex-emotions course, perception index of life-sex-emotions education, life value, sex

## Abstract

**Aim:** Adolescence is a time of transition from childhood to adulthood, when young people go through a number of vital physical and psychological developments. It is surprising yet unfortunate that the number of teenage suicide deaths and teenage infections of gonorrhea have increased over the years, becoming serious public health concerns in Taiwan. The aim of this study was to investigate the effect of an education course on teenagers' understanding of adolescence and their attitudes toward life, sex, gender equality, and mental health.

**Material and Methods:** Participants were comprised of Taiwanese students in Grades 5 to 9 who completed a Life–Sex–Emotions course, titled “Sailing through Adolescence.” The effect of the course was measured using pre- and post-test scores on the Perception Index of Life–Sex–Emotions Education (PILSEE) instrument. Qualitative data included subjective responses to questions before and after the course. Data were collected between September 2017 and June 2020.

**Results:** A total of 10,506 completed questionnaires were collected. The mean PILSEE pretest scores for each subscale ranged from 8.71 to 13.37 (SD = 1.499–1.99); posttest subscale scores ranged from 9.30 to 13.95 (SD range = 1.490–2.288). The mean overall pretest score was 86.86 (SD = 10.83); the mean posttest score was 92.62 (SD = 10.30). The paired *t*-test demonstrated that post-test scores were significantly higher than pretest scores (*t* = 55.46; *p* < 0.01). Qualitative feedback indicated that the course improved students' self-esteem, their understanding adolescence, and awareness of influences of the media.

**Conclusion:** Our findings indicate that an educational course about life, sex, and emotions during adolescence can be an effective intervention to help teenagers understand the impact of adolescence on attitudes toward life, sex, mental health, and gender equality.

## Introduction

Adolescence is a significant transitional stage between childhood and adulthood (1). Teenagers face many challenges during adolescence including the development of secondary sex characteristics, struggles between dependence and independence, hormone-related mood swings, annoying acne (2), and uncertainty about self-esteem and identity (3).

**Figure 1:**
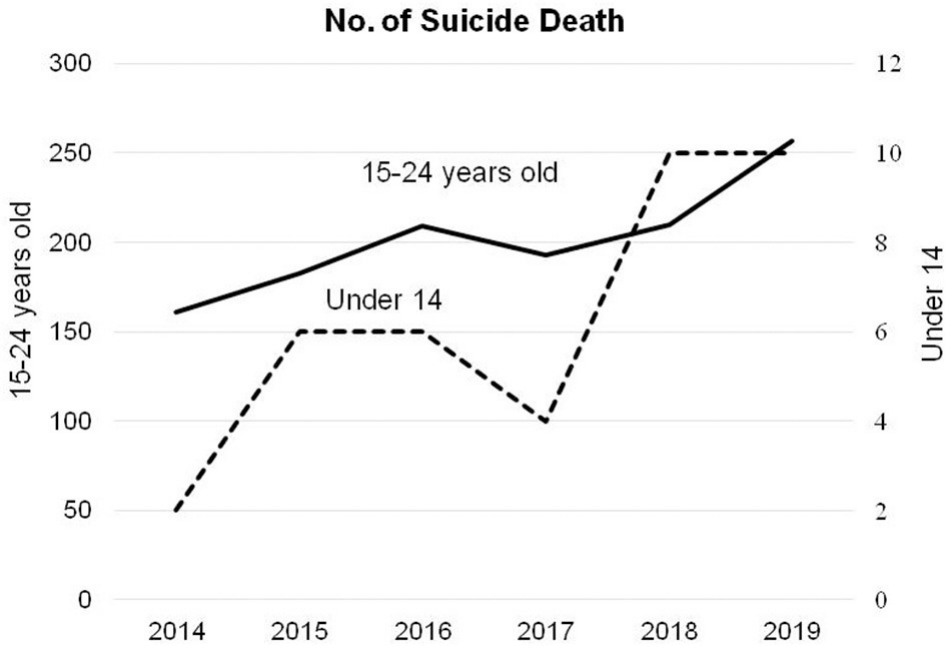
Suicide deaths in adolescents during 2014–2019 in Taiwan.

Teenage suicides have risen in Taiwan at an alarming rate. As shown in Figure 1, the number of suicide deaths among adolescents under the age of 14 years has increased from 2 to 10 per year between 2014 and 2019, and for adolescents between the ages of 15 and 24 years it increased from 161 to 257 during the same period (4). The suicide death rate of adolescents in 2019 reached a 10-year high. The common causes for teenage suicides in Taiwan were “a lack of meaning or value of life” and “to escape from or avoid pain” (5, 6).

**Figure 2:**
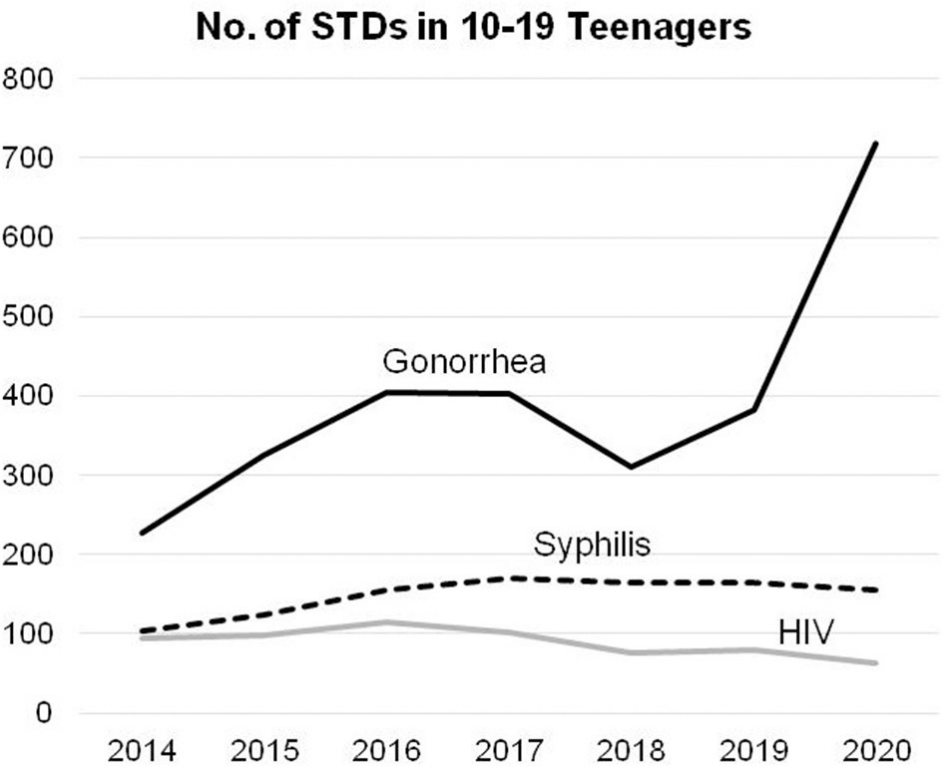
Teenage infections of syphilis, gonorrhea, and HIV during 2014–2020 in Taiwan.

Curiosity about sex is a natural and significant component of adolescence, which is often regarded as a topic that is taboo in Chinese society (7). Misconceptions of sex have also been spread by the media, increasing misunderstandings for teenagers and leading to infections from sexual transmitted diseases (STDs) and unwanted teenage pregnancies (8). Teenage infections with STDs have long been a concern in Taiwan, especially from gonorrhea (Figure 2) (9). In 2020, the number of teenagers infected with gonorrhea increased by almost 90%, yet the infection rate for adults only increased by 50% (9).

The importance of gender equality has become more significant, and social awareness has increased for adolescents. The Taiwanese government enacted the Gender Equity Education Act (GEEA) in 2004, which endeavors to promote gender equality (10). However, many of the changes that have been incorporated into textbooks have been considered by parents to be inappropriate for certain age groups. These have more recently been addressed by the Control Yuan, which is an independent investigatory and auditory agency of the government in Taiwan (11).

Issues considered to be important regarding the topic of sex education may differ between girls and boys. It was reported that boys showed positive interest in visits and discussions with nurses on sexual health (12) and sex life (13). In contrast, girls are likely to be more interested in topics of a responsible approach to sex life, sexual abuse, parenting, contraception, and gender equality (13).

To help teenagers smoothly transition through adolescence and develop positive and healthy attitudes toward life, sex, and gender equality, a course promoting Life–Sex–Emotions entitled “Sailing through Adolescence” has been offered in Taiwan since 2014 to students in grades 5 to 9. However, the impact of this course has not been quantitatively or qualitatively examined.

Therefore, the aim of this study was to investigate the effect of the “Sailing through Adolescence” course on perceptions of students in grades 5 to 9 regarding their understanding of adolescence and its impact on life, sex, emotions, and other associated issues. A pretest–posttest design examined students' perceptions using a Perception Index of Life–Sex–Emotions Education questionnaire (PILSEE) for quantitative measures and seven questions about students' subjective perceptions for qualitative measures.

## Methods

### Design

This retrospective cohort study used a pretest–posttest design to examine the effect of an educational course entitled “Sailing through Adolescence” on students' perceptions of life, sex, and emotions.

### Participants

Students in grades 5 to 9 enrolled in were selected by purposive sampling to ensure representation of diverse characteristics relevant to our study. Inclusion criteria for students were as follows: (1) enrolled in the “Sailing through Adolescence” course and (2) had demonstrated the ability to read and write. Exclusion criteria for students were (1) absent from four or more classes of the “Sailing through Adolescence” course and (2) had been identified as having a mental disorder. In this survey, sample size calculation for ±1% precision levels, a confidence interval of 95%, and *P* = 0.5 was determined to be 9,604 (14). Considering the 10% churn rate, 10,564 participants were needed.

### Ethical Considerations

This study was approved by the Ethics Committee of Taipei Hospital, Ministry of Health and Welfare (TH-IRB-0021-0001). All procedures performed were in accordance with the ethical standards of the Ethics Committee of Taipei Hospital, Ministry of Health and Welfare and with the 1964 Helsinki declaration and its later amendments or comparable ethical standards.

### The PILSEE Instrument

The 20-item PILSEE instrument was developed to assess students' attitudes about aspects of adolescent life. The PILSEE was designed by the same three experts that developed the education course and is comprised of seven subscales: value of Life (three items), Secrets of Adolescence (three items), Gender Roles (three items), Friendship and Love (three items), Myths of the Media (two items), Adolescent Sexuality (three items), and Views of Marriage and Sex (three items). Each item is scored on a Likert scale from 1 to 5; item scores range from 1 to 5. Subscale scores are the sum of the item scores; the total score ranges from 20 to 100. Higher scores indicate a more positive attitude toward adolescence. Data collected from students in 2014 (*n* = 8,693) examined validity and reliability of the scale. The face validity was 4.86–4.98; the CVI for content validity was good (81.67%). The Cronbach's alpha assessing for internal consistence for each of the seven subscales was from 0.733 to 0.898; the Cronbach's alpha for overall was 0.956. For confirmatory factor analysis, the factor loadings for 18 out of 20 questions were over 0.7, and the remaining two questions were 0.692 and 0.591. GFI was 0.953, AGFI was 0.933, and both NFI and NNFI were over 0.9. Overall, this questionnaire had good reliability and validity and could serve as an evaluation tool. Details of the PILSEE instrument are shown in Supplementary Table 1.

### The “Sailing Through Adolescence” Course

The “Sailing through Adolescence” course was offered in 12 weekly lessons over a period of 2–3 months and approved by the “Committee of School Curriculum Development” of all participating schools. The course was developed by three experts in sex education, gender equality education, and life-skills education for adolescents, which was based on cognition, affection, and behavior. Each lesson focuses on one specific topic. At the beginning of the course, students completed the PILSEE questionnaire for pre-test scores. Students were then presented with a “Treasure Map,” and stickers were pasted on areas of the map as a means of recording the contents of each lesson. A “Learning sheet” was also distributed to the students as a guide for reinforcing knowledge related to each lesson. Students summarized knowledge gained from each lesson with an activity called “Brother Parrot has something to say.” Students were encouraged to record and share their thoughts and impressions at the conclusion of each lesson. Details of the “Sailing through Adolescence” course are shown in Supplementary Table 2. After completing the 12 lessons, students filled out the PILSEE questionnaire for posttest data.

### Data Collection

Data were collected from 2017 to 2020. Pretest and posttest data were collected with the PILSEE questionnaire before and at the conclusion of the education course (Supplementary Table 2).

### Data Analysis

Quantitative data were analyzed using SPSS version 22.0 for Windows (Armonk, NY: IBM Corp). Descriptive statistics were used for frequency (n, %), and mean and standard deviations (SD) were used for total scores and subscale scores. Pair *t* tests assessed differences between pretest and posttest scores. Significance was set at *p* < 0.05 for statistical comparisons. Qualitative data were collected using from the learning sheets at the conclusion of each lesson.

## Results

### Participant Characteristics

A total of 10,558 students enrolled in the course from September 2017 to June 2020. However, 52 participants only completed 20% of the questions. Thus, pre- and post-test data were analyzed for 10,506 students. The remaining missing data was replaced by the mode. The sample loss rate was 0.49%. In terms of gender distribution of the participants, 5,402 were boys (51.4%) and 5,104 were girls (48.6%). In terms of age distribution, 10,200 (97.0%) were grade 5 to 6 pupils (aged 10–12) in elementary schools, and 306 (3.0%) were grade 7 to 9 pupils (aged 13–15) in junior high schools.

### PILSEE Scores

The mean PILSEE pretest scores for each subscale ranged from 8.71 to 13.37 (SD = 1.499–1.99); posttest subscale scores ranged from 9.30 to 13.95 (SD range = 1.490–2.288). The mean overall pretest score was 86.86 (SD = 10.83); the mean posttest score was and 92.62 (SD = 10.30). The paired *t* test indicated that posttest total scores and subscale scores were significantly higher than pretest scores (*p* < 0.001). Therefore, the significant improvement in posttest scores on the PILSEE suggests that the course on “Sailing through Adolescence” was effective in increasing students' attitudes toward adolescence. Details are shown in Table 1.

**Table 1:** Pretest and posttest scores for students and differences between scores on the PILSEE (*N* = 10,506).

	**Pre-test**	**Post-test**		
	**Mean ± SD**	**Mean ± SD**	***t***	***P***
Total PILSEE score (range = 20–100)	86.86 ± 10.83	92.62 ± 10.30	55.46^***^	<0.001
**PILSEE subscale scores**				
Value of life (range = 3–15)	12.69 ± 2.25	13.83 ± 1.90	54.28^***^	<0.001
Secrets of adolescence (range = 3–15)	12.86 ± 2.28	13.89 ± 1.86	44.75^***^	<0.001
Gender roles (range = 3–15)	13.37 ± 1.99	13.94 ± 1.77	28.56^***^	<0.001
Friendship and love (range = 3–15)	13.33 ± 1.90	13.95 ± 1.73	31.47^***^	<0.001
Myths of the media (range = 2–10)	8.71 ± 1.49	9.30 ± 1.22	38.30^***^	<0.001
Adolescent sexuality (range = 3–15)	13.00 ± 2.15	13.91 ± 1.79	40.43^***^	<0.001
Views of marriage and sex (range = 3–15)	12.89 ± 2.13	13.80 ± 1.84	41.83^***^	<0.001

*SD, standard deviation*.

*** *p < 0.001*.

#### Difference Between Pre-test and Post-test PILSEE Scores for Boys and Girls

To examine if there were any differences in improvements in PILSEE scores between boys and girls following completion of the “Sailing through Adolescence” course, we used the difference between the mean posttest and pretest scores (Table 2). Boys scores were significantly greater for the total score (*p* < 0.01) as well as the subscales for Secrets of Adolescence (*p* < 0.001), Gender Role (*p* < 0.05), and Friendship and Love (*p* < 0.01). These findings indicate that the education course had a more significant effect on boys' attitudes toward adolescence in these three areas.

**Table 2:** Amount of improvement between pretest and posttest for total score and subscale scores for boys (*n* = 5,402) and girls (*n* = 5,104) in grades 5–9 on the PILSEE.

	**Boys**	**Girls**		
	**Mean ± SD**	**Mean ± SD**	***t***	***P*-value**
Total score	6.02 ± 11.98	5.49 ± 9.01	2.60^**^	0.009
**Subscale scores**				
Value of Life	1.15 ± 2.33	1.14 ± 1.96	0.25	0.805
Secrets of adolescence	1.17 ± 2.59	0.87 ± 2.03	6.52^***^	<0.001
Gender roles	0.62 ± 2.26	0.52 ± 1.78	2.53^*^	0.011
Friendship and love	0.68 ± 2.25	0.55 ± 1.72	3.32^**^	0.001
Myths of the media	0.58 ± 1.69	0.60 ± 1.45	−0.73	0.468
Adolescent sexuality	0.91 ± 2.46	0.90 ± 2.13	0.29	0.771
Views of marriage and sex	0.91 ± 2.44	0.91 ± 1.99	0.21	0.836

*SD, standard deviation*.

* *p < 0.05*,

** *p < 0.01*,

*** *p < 0.001*.

#### Difference Between Pre-test and Post-test PILSEE Scores for Elementary School and Junior High School Students

The overall score of improvement for elementary pupils was 5.80 points (SD = 10.66), and that for junior high school students was 4.59 (SD = 10.00), which did not differ significantly between the two cohorts. However, two subscale scores increased significantly more for elementary school students: secrets of Adolescence (*t* = 2.88, *p* < 0.01) and Views of Marriage and Sex (*t* = 2.68, *p* < 0.01) (Table 3).

**Table 3:** Difference in pretest and posttest scores on the PILSEE between elementary school students (*n* = 10,200) and junior high school students (*n* = 306).

	**Elementary**	**Junior high**		
	**Mean ± SD**	**Mean ± SD**	***t***	***P*-value**
Total score	5.80 ± 10.66	4.59 ± 10.00	1.95	0.051
**Subscale scores**				
Value of life	1.15 ± 2.17	1.03 ± 1.91	0.91	0.364
Secrets of adolescence	1.03 ± 2.35	0.71 ± 1.89	2.88^**^	0.004
Gender roles	0.57 ± 2.05	0.42 ± 1.68	1.55	0.121
Friendship and love	0.62 ± 2.02	0.64 ± 1.91	−0.20	0.844
Myths of media	0.59 ± 1.58	0.50 ± 1.50	0.97	0.331
Adolescent sexuality	0.92 ± 2.31	0.65 ± 2.12	1.96	0.050
Views of marriage and sex	0.92 ± 2.24	0.62 ± 1.88	2.68^**^	0.008

*SD, Standard deviation*.

** *p < 0.01*.

### Qualitative Findings

Qualitative and descriptive studies allow researchers to gain an understanding of the experiences of participants closer to the truth (15). We also collected some anonymous feedback on the seven subscales for further analyses. This feedback about the seven subscales suggested that students' self-esteem, understanding of adolescence, the role of gender, the ability to analyze the influence of the media, and sexuality all improved after the education course.

Two students provided the following response to their personal perspective on *Value of Life*. One student (Student A) said, “I learned what sex is, and I also understood myself better. I am willing to accept who I am. I will not care about others' comments and I will live for myself.” Student B offered the following perspective:

*The course is over, but I wish I could take it again. I am particularly impressed by the lesson of “Rainbow Island.” I learned that our lives are meaningful and useful. It is just like everything in nature. I used to think my life was worthless when I encountered difficulties, but I've learned to cherish my precious life*.

Adolescence is a period for a person to go through dramatic changes, both inside and outside. There are physical, psychological, and emotional changes of young persons during this time. Two students provided the following feedback on *Secrets of Adolescence*. Student C stated, “There are physical changes of boys and girls in adolescence, and I think it's important to know about these changes. I find this course useful, and I will concentrate more on this course,” while Student D said,

*I think this course has helped me understand myself better as well as the changes during adolescence, and how to prepare for them. It also helped me to know that those changes are normal and there's nothing wrong with me. We should be thankful that we are healthy. We must cherish and accept ourselves, and we need to be more confident and accept challenges in adolescence and in life*.

The importance of not having specific expectations for behaviors that are associated with gender was an important lesson for Student E, who shared the following feedback about one of the videos:

*The boy liked to play house so his classmates bullied and laughed at him in the film. He was very unhappy and sad. However, his teacher found that he was a responsible boy and would help his mother. We should not have gender stereotypes of traits or hobbies. For example, my dad is a sushi chef and a cook. We should respect the hobbies and traits of others*.

Friendship and love are vital relationships that people cultivate and cherish in their lives and are some of the most valuable treasures a person can possess. Student G said, “Love is like sailing a boat. It is fun but can be dangerous,” while a Student F shared the following:

*I think confession should be from one's heart and should be sincere. We should not judge others by their looks, but instead, we shall focus on the beauty of peoples' personalities. We should not make friends with people only for their good looks, as some good-looking people could be rude. There are many kinds of people on the Internet. Don't talk to anyone you are not familiar with on the internet, because you don't know him/her quite well*.

Myths of the Media is the force exerted by the media, resulting in either a change of or reinforcement of beliefs, views, and perceptions in audience. Student H had the following opinion:

*Everyone has a mobile phone today. We should learn how to distinguish all the online information. There are some pornographic, exaggerated and shocking advertisings on TV, and they are not worth learning about. Therefore, it's a must to learn how to filter unwanted and misleading information in the media in order to grow up healthily. We should be cautious in this diverse society*.

Adolescent sexuality is a stage of human development when adolescents experience and explore sexual feelings (16). Student I had a strong opinion about this subscale:

*It is not proper to have sex when we are teenagers. We could scream or run into shops for help if we encounter sexual harassment or assaults. The most important thing is to protect ourselves and be safe. Each of us is unique. We should work hard and never give up*.

Marriage is a long-term commitment to spend your life with someone. Two students provided the following feedback:

*You must think twice before doing things so that you will not regret. We shall do the right things rather than do the bad things. (You should have sex) only if you really want to have children. I saw news about many children losing lives due to child abuse, and this really made me very sad*. (Student J)

*In marriage, I will not leave my husband for someone richer or better. I just want to love my husband and accompany him through all the difficulties*. (Student K)

## Discussion

Our study results showed that the “Sailing through Adolescence” course for Life–Sex–Emotions was effective for improving the perceptions of students in grades 5 to 9 about adolescence, attitudes toward life, sex, mental health, and gender equality. These attitudes might also enhance adolescents' self-awareness, attitudes toward physical changes during adolescence, and sexual health.

Regarding the prevention of teenage suicides, the Ministry of Education (MOE) in Taiwan established the “Three-level Prevention Program for Students Self-harm on Campus” (17). The first-level prevention is enhancing students' mental health through courses to protect students from self-harm. The second-level prevention is about early detection of students of high suicide risks and offering intervening counseling. The third level of prevention is to establish a crisis management system, which combines community resources to prevent further incidents of suicide. The course of “Sailing through Adolescence” improved students' self-esteem, which might also be included in first-level prevention of teenage suicide.

Regarding the increasing cases of STD among teenagers, it is vital to have quality sex education, so that it would help young people understand how pressure from family, peer, and media could impact their health (18). They would also then learn how to access valid and reliable health information about HIV/STDs and to make informed decisions about their health (19). As to gender equality, the goal of the course was to promote awareness of gender equality and eliminate gender discrimination (20). This course was therefore effective in sexual education and gender equality education according to participants' responses.

Topics such as “Secrets of Adolescence,” “Friendship and Love,” and “Gender Roles” would be more interesting for the boys, which were similar to the findings of other groups that boys would discuss sex life the most (13). Therefore, the strategies of the sex education should be more flexible in teaching boys and girls.

The most exciting message of the feedback was that the teenagers learned that their lives are precious and meaningful, and they should not give up no matter how hard the life is. They also learned the physical, psychological, and emotional changes caused by hormone changes in the adolescence; the sexual knowledge and protection in sexual relationships and activities; the alertness about media; and the responsibilities in the marriage from this course.

In addition, the effects of this course could also be compared to the results of other studies on improving cognition. For example, in one study about children's cognitions of HIV, only 17.85% of children before taking the lesson “Sex Island” (one lesson in this course) answered HIV-related questions correctly, yet from the lesson, children learned that “they would not be infected with HIV if they ate, hugged or swam with HIV carriers” in the lesson (21).

The “Sailing through Adolescence” course incorporated interactions of affect, behavior, and cognition (ABC) (22) in order to improve students attitudes toward adolescence. Affective states may also influence the decision-making process even when the source of the affect is not directly related to the choices under evaluation. Incidental affects also impact on perception, memory, judgment, and behavior (23). Therefore, the affective states may influence the teenagers' behavioral strategies.

## Limitations

The results of our study are limited by the lack of a control group, which would help reduce confounding biases. Additional studies should compare scores on the PILSEE for students in similar grades who did not enroll in the course on “Sailing through Adolescence.” This course was introduced to schools in 2014, and the validity or reliability of the questionnaire was not fully accomplished at that time. Due to the increasing attention to these issues from teachers and parents over the years, the retrospective validity and reliability of the questionnaire were amended, and some statistics were not measured. The last two questions of PILSEE were slightly revised in the new version after 2017. Although this course has potential to improve teenagers' attitudes toward adolescence, the course has only been offered for 6 years. A longitudinal study with follow-ups in adulthood will be needed to better assess the effects of “Sailing through Adolescence.”

## Data Availability Statement

The raw data supporting the conclusions of this article will be made available by the authors, without undue reservation.

## Ethics Statement

The ‘‘Sailing through Adolescence'' course was approved by the ‘‘Committee of School Curriculum Development'' of all participating schools, and was supervised by the teachers. The studies involving human participants were reviewed and approved by Ethics Committee of Taipei Hospital, Ministry of Health and Welfare (TH-IRB-0021-0001) in accordance with the national legislation and the institutional requirements.

## Author Contributions

K-YC collected the data and processed further analyses. WC conceived the present idea, wrote the manuscript, and took primary responsibility for communication with the journal and editorial office during the submission process, throughout peer review, and during publication. Both authors contributed to the article and approved the submitted version.

## Conflict of Interest

The authors declare that the research was conducted in the absence of any commercial or financial relationships that could be construed as a potential conflict of interest.

## References

[1] Sawyer SM Azzopardi PS Wickremarathne D Patton GC. The age of adolescence. Lancet Child Adolesc Health. (2018) 2:223–8. 10.1016/S2352-4642(18)30022-1 30169257

[2] Revol O Milliez N Gerard D. Psychological impact of acne on 21st-century adolescents: decoding for better care. Br J Dermatol. (2015) 172 (Suppl. 1) 52–8. 10.1111/bjd.13749 25702715

[3] Minev M Petrova B Mineva K Petkova M Strebkova R. Self-Esteem in Adolescents. Trakia J Sci. (2018) 16:114–8. 10.15547/tjs.2018.02.007

[4] Ministry of Health and Welfare Taiwan. National Statistics of Suicide Death Rate. (2020). Available online at: https://dep.mohw.gov.tw/domhaoh/fp-4904-8883-107.html (accessed January 28).

[5] Wang SH. Causes of juvenile suicide behaviors and prevention strategies. J Taiwan Educ Rev. (2016) 5:160–4. (Chinese). Available online at: http://www.ater.org.tw/journal/article/5-12/free/24.pdf

[6] Stewart JG Shields GS Esposito EC Cosby EA Allen NB Slavich GM Auerbach RP. Life stress and suicide in adolescents. J Abnorm Child Psychol. (2019) 47:1707–22. 10.1007/s10802-019-00534-5 31028559 PMC6717522

[7] Wong WC Lee A Tsang KK. Correlates of sexual behaviors with health status and health perception in Chinese adolescents: a cross-sectional survey in schools. AIDS Patient Care STDS. (2004) 18:470–80. 10.1089/1087291041703656 15321018

[8] Landry M Turner M Vyas A Wood S. Social Media and Sexual Behavior Among Adolescents: Is there a link? JMIR Public Health Surveill. (2017) 3:e28. 10.2196/publichealth.7149 28526670 PMC5457530

[9] Taiwan Centers for Disease Control. Taiwan National Infectious Disease Statistics System. (2020). https://nidss.cdc.gov.tw/en/nndss/disease?id=098 (accessed January 28).

[10] Ministry of Education Taiwan. Gender Equity Education Act (GEEA). (2018) https://law.moj.gov.tw/Eng/LawClass/LawAll.aspx?PCode=H0080067 (accessed January 28).

[11] The Control Yuan Taiwan. Committee Member Fehng-Shian Gau Corrected the Film “Shall We Swim?” Which was Subsidized by the Ministry of Interior. (2014). https://www.cy.gov.tw/CyBsBoxContent.aspx?n=133&s=3916 (accessed January 28).

[12] Dittus PJ Harper CR Becasen JS Donatello RA Ethier KA. Structural intervention with school nurses increases receipt of sexual health care among male high school students. J Adolesc Health. (2018) 62:52–8. 10.1016/j.jadohealth.2017.07.017 29102554 PMC6739836

[13] Pavelová L Archalousová A Slezáková Z Zrubcová D Solgajová A Spáčilová Z. The need for nurse interventions in sex education in adolescents. Int J Environ Res Public Health. (2020) 18:492. 10.3390/ijerph18020492 33435342 PMC7827239

[14] Israel GD. Determining Sample Size. University of Florida: IFAS extension, PEOD-6 (1992). https://www.researchgate.net/profile/Subhash_Basu3/post/What-is-the-minimum-sample-size-for-online-survey/attachment/5ec41296ead4db0001569d28/AS%3A892953522364416%401589908118112/download/samplesize1.pdf

[15] Sandelowski M. What's in a name? Qualitative description revisited. Res Nurs Health. (2010) 33:77–84. 10.1002/nur.20362 20014004

[16] Kar SK Choudhury A Singh AP. Understanding normal development of adolescent sexuality: a bumpy ride. J Hum Reprod Sci. (2015) 8:70–4. 10.4103/0974-1208.158594 26157296 PMC4477452

[17] Ministry of Education Taiwan. Three-Level Prevention Program for Students Self-harm on Campus. (2014). Available online at: https://depart.moe.edu.tw/ed2800/News_Content.aspx?n=29D1A6CC2883568E&sms=CD00C8B5422B5957&s=4E789772C47F2094 (accessed January 28).

[18] Mollborn S Lawrence E. Family, peer, and school influences on children's developing health lifestyles. J Health Soc Behav. (2018) 59:133–50. 10.1177/0022146517750637 29298103 PMC5898799

[19] Centers for Disease Control and Prevention USA. What Works: Sexual Health Education. (2020). Available online at: https://www.cdc.gov/healthyyouth/whatworks/what-works-sexual-health-education.htm (accessed January 28).

[20] CEDAW. Guidelines of Convention on the Elimination of All Forms of Discrimination against Women. (2008). Available online at: https://www.un.org/womenwatch/daw/cedaw/ (accessed January 28).

[21] Wu CJ. A study on learning effectiveness of applying rainbow life education curriculum in gender equality education for the sixth grade students in elementary school (dissertation/master's thesis). Shih Hsin University, Taipei, Taiwan (2017). (Chinese)

[22] Ostrom TM. The Relationship between the affective, behavioral, and cognitive components of attitude. J Exp Soc Psychol. (1969) 5:12–30. 10.1016/0022-1031(69)90003-115362425

[23] Winkielman P Knutson B Paulus M Trujillo JL. Affective influence on judgments and decisions: moving towards core mechanisms. Rev Gen Psychol. (2007). 11:179–92. 10.1037/1089-2680.11.2.179

