# Supplementary material for: An Effective Life–Sex–Emotions Course for Taiwanese Adolescents on Health Perceptions: A Cohort Study
Source: Front Public Health. 2021 May 19;9:660229. doi: 10.3389/fpubh.2021.660229 (PMC8170017; doi:10.3389/fpubh.2021.660229)
Supplement: Supplementary file 1 [file Data_Sheet_1.docx]

**Supplementary Table 1S** Perception Index of Life-Sex-Emotions Education (PILSEE) Instrument

| **Subscale** | **Items** | **Strongly Agree** | **Agree** | **Neither Agree nor Disagree** | **Disagree** | **Strongly Disagree** |
| --- | --- | --- | --- | --- | --- | --- |
| **Value of Life** | 1. I am unique and valuable, and I cherish my life. | 5 | 4 | 3 | 2 | 1 |
|  | 2. I can accept everything about myself, including my gender, strengths, and weakness. | 5 | 4 | 3 | 2 | 1 |
|  | 3. I know my dreams and goals in life and will overcome obstacles to achieve them. | 5 | 4 | 3 | 2 | 1 |
| **Secrets of Adolescence** | 4. I understand the changes in my body in adolescence. | 5 | 4 | 3 | 2 | 1 |
|  | 5. I am positive about the changes in my body in adolescence. | 5 | 4 | 3 | 2 | 1 |
|  | 6. I know how to deal with the changes in my body in adolescence. | 5 | 4 | 3 | 2 | 1 |
| **Gender Roles** | 7. I can accept and respect people of other genders and different traits. | 5 | 4 | 3 | 2 | 1 |
|  | 8. I don’t judge others by gender stereotypes. | 5 | 4 | 3 | 2 | 1 |
|  | 9. Everybody has equal rights and deserves respect regardless of gender. | 5 | 4 | 3 | 2 | 1 |
| **Friendship and Love** | 10. I know how to get along with classmates of the same and opposite sex and have healthy friendships. | 5 | 4 | 3 | 2 | 1 |
|  | 11. I cherish my feelings and do not rush to fall in love. | 5 | 4 | 3 | 2 | 1 |
|  | 12. I know how to say no to sexual harassment and avoid traps when making friends online. | 5 | 4 | 3 | 2 | 1 |
| **Myths of the Media** | 13. Messages about ‘sex’ and ’love’ in the media are often exaggerated and wrong. | 5 | 4 | 3 | 2 | 1 |
|  | 14. I can distinguish and reject negative messages in the media. | 5 | 4 | 3 | 2 | 1 |
| **Adolescent Sexuality** | 15. I know that during adolescence it is common to have sexual feelings. | 5 | 4 | 3 | 2 | 1 |
|  | 16. I know how to cope with my sexual feelings properly. | 5 | 4 | 3 | 2 | 1 |
|  | 17. I can protect myself and avoid being sexually violated. | 5 | 4 | 3 | 2 | 1 |
| **Views of Marriage and Sex** | 18. I think marriage is important and I’m looking forward to getting married when I am an adult. | 5 | 4 | 3 | 2 | 1 |
|  | 19. I think teenagers should avoid having sex. | 5 | 4 | 3 | 2 | 1 |
|  | 20. I will not have sex in my teenage years. | 5 | 4 | 3 | 2 | 1 |

**Supplementary Table 2S** Topics covered during Sailing through Adolescence using the Treasure Map

| **Week** | **Treasure Map** | **Focus of the session** |
| --- | --- | --- |
| 1 | “Youth” Boat | Prepare students for changes during adolescence and provide guidance for how to seek support when problems are encountered. |
| 2 | “Cherish” Island | Provide information to help students understand that everyone is unique and valuable and should be valued. |
| 3 | “Body” Town | Provide information about physical and psychological changes associated with gender during adolescence, and help students accept their own unique development. |
| 4 | “Rainbow” Island | Encourage students to articulate their dreams and goals; present skills that can help them overcome obstacles. |
| 5 | “Phantom” Castle | Provide information to students about gender stereotypes in order to increase students’ respect for gender differences. |
| 6 | “Bubbling” Island | Provide information to help students distinguish the difference between love, passion and infatuation. Describe how to prepare for and develop interpersonal relationships, rather than rushing into romantic relationships. |
| 7 | “Media” Park | Provide information on how the media can affect students’ values in order to help them recognize negative information in the media. |
| 8 | “Sex” Island | Provide information on the value and meaning of sex and sexual feelings during adolescences, in order to help students avoid chaotic sexual relationships. |
| 9 | “Pregnancy” Village | Provide information on the consequences of adolescent pregnancy and the importance of contraception. |
| 10 | “Protection” Peninsula | Provide information to help students understand how to protect themselves in relationships, how to reject sexual harassment, and how to deal with online dating. |
| 11 | “Marriage” Bay | Provide information to help students to prepare themselves for a happy marriage as adults. |
| 12 | “Wait” City | Discuss any questions or concerns students have from the previous lessons. Help students understand the importance of waiting. |
